# Therapeutical Notes on Cascara Sagrada

**Published:** 1890-08

**Authors:** C. E. Murphey

**Affiliations:** Atlanta, Ga.


### THERAPEUTICAL NOTES ON CASCARA SAGRADA.

BY C. E. MURPHEY, M. D., ATLANTA, GA.

Quite a discusssion came up a few weeks ago among the members of the Atlanta Society of Medicine as to the value of cascara sagrada as a drug, and I was somewhat surprised to find so many reports of bad results from this agent which is so valuable in constipation and disorders that arise from a sluggish state of the bowels. Some of the physicians present expresed themselves as having discarded its use altogether, on account of failure to get any satisfactory results whatever. I consider cascara sagrada one of the most valuable laxatives in the materia medica. I do not give cascara sagrada for its purgative effect. Its local action on the intestine canal and its effect on the nervous system show that it should not be used as a purgative, as there are many other drugs that are better suited as purgatives.

In laxative doses you will never get any evil results, but will return healthy action of the entire alimentary canal. It has a mild stimulating effect on the liver, aids digestion, increases the gastro-intestinal secretion and favors nutrition generally. But there are two important facts to remember. First, quality; second, the dose.

There have, doubtless, been placed on the market by many of our drug houses inferior articles of cascara sagrada; therefore scores of physicians have been disappointed in obtaining good results, and give adverse reports as to its true therapeutic virtues. Its scarcity and the expense of preparation have lead some manufacturers to prepare other combinations, with a hope of producing similar effects to the pure article. Thus we often are getting the effects of aloes, strychnine, podaphyllin and other combinations instead of cascara sagrada, and here the cry of failure comes from those who have been so unfortunate as to obtain this inferior quality. During the time I handled drugs I found that I could buy cascara sagrada as low as one dollar and twenty-five cents per pound, when a pure article was worth three dollars; from this alone you can imagine how uncertain the action may be unless you can obtain the pure and reliable goods. I have obtained best results from Parke, Davis & Co.'s Extract and Fluid Extract. Unless you specify the manufacturer your prescription may be filled with an inferior article.

DOSE.—To determine the dose of cascara sagrada is of great importance. I never give cascara sagrada except for its laxative effect. The dose must be determined by the results and close observation. In uncomplicated cases of constipation, I use the following formula:

℞. Ext. cascaræ sagradæ..............................f℥ j.

Glycerini........................................℥ss.

Syr. Tolut.......................................℥ss.

Aquæ, q. s.......................................℥iv.

Sig.—Teaspoonful in water twice daily before meals; after taking a few days reduce to half dose.

Or have used this combination, especially if patient suffers from indigestion:

℞. Ext. cascaræ sagradæ......................f℥ss.

Tr. nucis vom.............................ʒiij.

Tr. rhus. tox.........................gtt. xv.

Syr. tolut...................................℥ j.

Aquæ, q. s...................................℥iv.

Sig.—Teaspoonful before meals in water. (M.)

I have used cascara sagrada in treatment of acute and chronic rheumatism. In acute cases I combine salicylate of soda, and in chronic cases Tr. chloride of iron. In acute cases I generally use this formula:

℞. Sodii salicylat...........................ʒiv.

Ext. cascaræ sagrad.......................ʒiv.

Ext. pep..................................ʒss.

Glycerini.................................℥ss.

Aquæ, q. s................................℥iv.

Sig.—Teaspoonful every three hours first day; afterwards every four hours.

Or in chronic cases:

℞. Ext. cascaræ sagrad........................℥ss.

Tr. ferri chlor.........................f℥jss.

Syr. tolut...........................℥jss.

Aquæ, q. s...........................℥iv.

Sig.—Teaspoonful in water four times daily. Remember in all cases where you get more than laxative effect to reduce the dose. I find in many cases after the use of cascara sagrada for a few days we are compelled to reduce the dose, as it will begin to act too freely. In other cases you will have to persist in its use for a few days before you get the laxative effect desired.

I find it valuable in hemorrhoidal tumors, and useful in pelvic congestions by relieving the engorged state of the blood vessels.

A member of the Atlanta Society of Medicine stated that he had been advised not "to give cascara sagrada in pregnant women, as there was danger of producing abortion." I never use it in purgative doses, and don't think it advisable to do so from the history of its action, especially in pregnant women. But this evil effect may be due to an inferior article adulterated with aloes. You can use it in laxative dose in pregnant women with best results.

